# Fabrication of Semiconducting Methylammonium Lead Halide Perovskite Particles by Spray Technology

**DOI:** 10.1186/s11671-017-2430-0

**Published:** 2018-01-10

**Authors:** Mohammad-Reza Ahmadian-Yazdi, Morteza Eslamian

**Affiliations:** University of Michigan-Shanghai Jiao Tong University Joint Institute, Shanghai, 200240 China

**Keywords:** Perovskite solar cells, Perovskite particles, Perovskite nanocrystals, Droplet-to-particle, Spray pyrolysis

## Abstract

In this “nano idea” paper, three concepts for the preparation of methylammonium lead halide perovskite particles are proposed, discussed, and tested. The first idea is based on the wet chemistry preparation of the perovskite particles, through the addition of the perovskite precursor solution to an anti-solvent to facilitate the precipitation of the perovskite particles in the solution. The second idea is based on the milling of a blend of the perovskite precursors in the dry form, in order to allow for the conversion of the precursors to the perovskite particles. The third idea is based on the atomization of the perovskite solution by a spray nozzle, introducing the spray droplets into a hot wall reactor, so as to prepare perovskite particles, using the droplet-to-particle spray approach (spray pyrolysis). Preliminary results show that the spray technology is the most successful method for the preparation of impurity-free perovskite particles and perovskite paste to deposit perovskite thin films. As a proof of concept, a perovskite solar cell with the paste prepared by the sprayed perovskite powder was successfully fabricated.

## Background

Various forms of the organometal halide perovskites utilizing various cations, such as methylammonium (MA), formamidinium (FA), cesium (Cs), or a combination of thereof, are very attractive photovoltaic materials and are currently widely explored to develop conventional thin-film perovskite solar cells, e.g., [1–4], as well as flexible and low weight to power [5] and tandem perovskite-based solar cells [6]. MA and FA cations are organic, less stable, and cheaper than Cs, which is a rare metal. While the majority of the research activities on the perovskites focus on thin-film solar cells, such molecular semiconductors could play a role in other similar fields, such as field effect transistors [7], perovskite light-emitting diodes [8], and high-energy radioactive radiation sensors [9].

In most perovskite-based devices, the perovskites are directly deposited in the form of thin films. However, several recent works have reported the fabrication of the perovskite semiconductors in the nanocrystal or particulate form. Perovskite nanocrystals exhibit high photoluminescence quantum yields and quantum confinement effects, analogous to the conventional quantum dots, when their dimensions are reduced to sizes comparable to their respective exciton Bohr radii, bringing about new opportunities for the development of new devices [10–12]. Most of such studies are centered around all-inorganic Cs-based perovskites, owing to their higher stability, e.g., [13–30], followed by organic-inorganic MA-based perovskites, e.g., [31–41], and very few on the FA-based perovskites, e.g., [42]. Most of the abovementioned works have focused on the properties of the perovskite nanocrystals. Some works have fabricated perovskite devices such as perovskite light-emitting diodes that incorporate the nanocrystals in the form of thin films, e.g., [21, 27, 29]. Few works have proposed formulations to prepare perovskite inks such as inks containing lead halide nanocrystals mixed with MA precursors [41] for the deposition of the thin films for solar cell applications.

The perovskite nanocrystals with rather small sizes and controlled morphology, as reported by the abovementioned works, are commonly grown in the solution (wet chemistry) [11]. Schmidt et al. [31] prepared colloidal MAPbBr_3_ nanocrystals with the size of 6 nm by mixing the perovskite precursors with organic solvents. They also prepared homogeneous thin films of these nanoparticles by spin-coating. Hassan et al. [36] used a two-step solution method to prepare mixed MA-based perovskite nanodots, where first the lead halide seed particles form in the solution and then the MA solution is added in order to complete the process. All-inorganic Cs-based perovskite nanoparticles have been prepared using similar wet chemistry methods, such as injection of Cs precursors into the lead halide precursor solution containing hot, high boiling point solvents [30]. Most of the aforementioned works focus on the fabrication of perovskite nanocrystals, which show a quantum confinement effect. However, for most thin-film devices such as solar cells, the quantum confinement effect is immaterial, and the preparation of polycrystalline micro- and nano-perovskite particles and thin films with facile techniques is desirable.

## Presentation of the Hypothesis

In this work, we report the idea and successful preparation of MAPbI_3_ perovskite particles by low-cost and facile spray technology, for the first time. In this proposed method, following the well-known process of droplet-to-particle formation of pharmaceuticals and ceramics by spray drying and spray pyrolysis, e.g., [43–46], a spray nozzle atomizes the perovskite solution, where the droplets in the form of a mist are introduced into a single- or multi-stage hot wall (tubular) reactor. As the droplets travel along the reactor, the solvent evaporates, a chemical conversion occurs to convert the precursor droplets into the perovskite particles. Therefore, as a result of the presence of a chemical reaction, the process may be called spray pyrolysis. The produced perovskite particles are collected at the outlet of the reactor. The method is capable of producing small particles in the nanometer range, i.e., nanocrystals, if the solution is atomized using specialty atomization techniques, such as electrospray nozzles or low-concentration solutions [46]. In addition, the fragile as-prepared perovskite particles may break down to form nano-sized perovskite particles, to be elaborated on later in this paper.

**Fig. 1:**
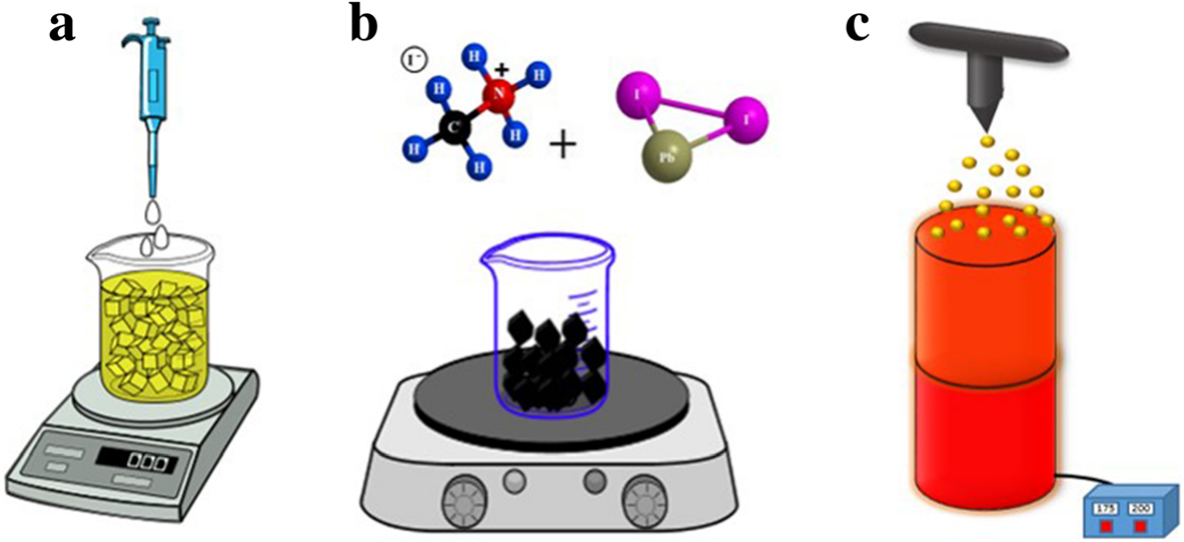
Three methods of producing perovskite powders used in this work: **a** wet chemistry anti-solvent method, **b** dry milling method (on a magnetic stirrer), and **c** droplet-to particle spray pyrolysis

In addition to the spray route, two other methods are proposed and tested for the preparation of the perovskite particles, i.e., the wet chemistry and milling. In the wet chemistry method, the perovskite precursor solution is added dropwise to an anti-solvent of the perovskite solvents, such as toluene, under stirring condition. The method leads to the precipitation of the perovskite particles in the anti-solvent. In the milling method, the perovskite precursors such as dry MAI and PbI_2_ powders are blended and milled, for instance in a hot plate magnetic stirrer, for several hours to react with one another, due to the mechanical forces. Figure 1 shows the schematic of the three proposed methods used in this work to prepare the perovskite particles.

## Testing the Hypotheses

In order to test the credibility of the proposed ideas, we have conducted preliminary experiments by preparing perovskite powders, as well as thin films and solar cells with the produced powders. Perovskite precursors were purchased from Xi’an Reagents Co., China, and other chemical were purchased from Sigma-Aldrich. The perovskite liquid solution used in the anti-solvent and spray methods were prepared by dissolving 158 mg of MAI and 420 mg of PbI_2_ powders in 1 ml of dimethyl sulfoxide (DMSO).

**Fig. 2:**
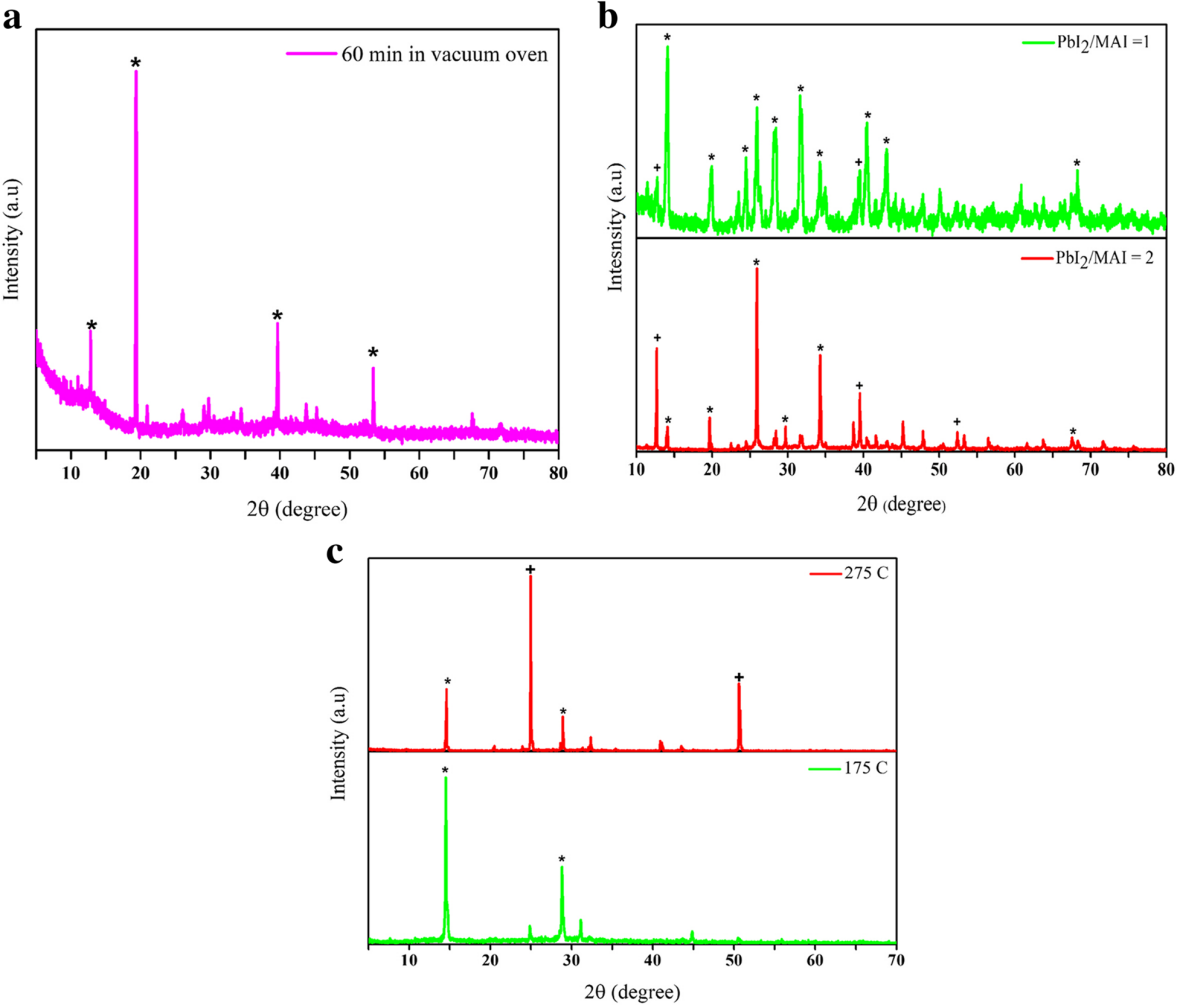
XRD pattern of perovskite powder prepared by **a** anti-solvent method, **b** milling method at two PbI_2_/MAI mass ratios, and **c** spray method when the temperature of the second heater (second stage of heating) is kept at two different temperatures of 175 and 275 °C, while the first heater (first stage of heating) is kept at 275 °C in both cases. The asterisk denotes the perovskite peaks

(i) Anti-solvent method
  In the anti-solvent method, the perovskite solution was added to toluene dropwise under stirring condition. After 2 min, yellow perovskite powder precipitated at the bottom and sidewalls of the beaker, and after 20 min of stirring, colloidal perovskite powder was observed in toluene, as well. This product (after 20 min) was annealed in an oven at 150 °C for 60 min. Figure 2a shows the X-ray diffraction (XRD; model D5005, Bruker, Germany) of the perovskite powder prepared by an anti-solvent method, where it is evident that the precursors have converted to the perovskite, although some weak peaks, associated with impurities are present.
(ii) Milling method
  Testing the idea of blending and milling of the dry perovskite precursors for the preparation of the perovskite powder requires a well-designed milling machine to provide sufficient forces. Here, in order to test the idea, a simple hot plate magnetic stirrer was used. The MAI and PbI_2_ powders were mixed with the mass ratios of PbI_2_/MAI of 1 and 2. The hot plate was kept at 200 °C, and the dry powders were blended and crushed in the container due to the force of the magnetic stirring bar. In wet chemistry preparation of perovskite precursor solution, the mass ratio of PbI_2_/MAI is around 3 (as mentioned above for the preparation of the perovskite solution), whereas in the milling method, we found that lower mass ratios (less PbI_2_ than stoichiometric) is more effective, in that the reaction of the precursor powders and conversion to the perovskite is improved. Figure 2b shows the XRD patterns of the produced perovskite powder for the PbI_2_/MAI mass ratios of 1.0 and 2.0. In general, the mass ratio of 1.0 is more successful in producing the perovskite powders; however, traces of impurities are present. This may be due to the insufficient interacting forces between the two precursors that results in traces of the initial precursors mixed up with the perovskite powder. Therefore, the milling approach was not successful in producing pure perovskite structure. Using a well-designed milling machine and careful control of the process parameters, such as the milling time and temperature, and addition of small amount of proper solvents to facilitate the process may improve the purity and the crystalline structure of the powders.
(iii) Spray method
  In the spray method, the perovskite solution was atomized with an air-assisted spray nozzle with a nozzle diameter of 0.2 mm, where the air pressure was set to 2.0 psig. The spray droplets were introduced into two vertically stacked stainless steel tubular heaters with a diameter of 10 cm, a length of 30 cm, with the maximum power of 800 W, each (Yancheng Huabang Electric Equipment Co., Ltd). The first heater was kept at 275 °C, so as to quickly evaporate the solvent, and the second or bottom heater was kept at either 275 °C or a lower temperature of 175 °C, where the latter was used to avoid the decomposition of the perovskite powders that had already formed. As Fig. 2c shows, the powder produced when the temperature of both heaters is kept at 275 °C contains high intensity peaks of PbI_2_, whereas when the temperature of the second heater is reduced to 175 °C, the impurities are nearly disappeared and the crystallinity of the perovskite is increased. In summary, the XRD results of the powders produced using the three abovementioned methods (Fig. 2) substantiate the merit of the spray method for producing pure and crystalline perovskite powders.

**Fig. 3:**
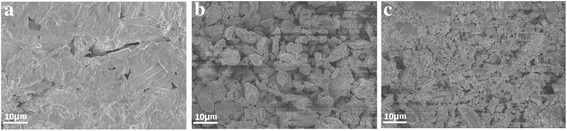
SEM images of the powders produced by **a** anti-solvent, **b** milling, and **c** spray methods

Figure 3 shows the scanning electron microscope (SEM; Hitachi, Model S-3400N) images of the produced powders by the three aforementioned methods. It is observed that the collected powders are somewhat agglomerated, which may have happened during the preparation or analysis. Nevertheless, the images of the powders prepared by the milling and spraying show the shape and size of the individual particles. The particles are few microns in size and have non-spherical and irregular shapes. In the spray method, one may expect to see spherical particles, as each perovskite droplet usually dries to form a perovskite particle. The non-spherical shape may be due to the strong ionic forces within the droplets of perovskite and/or preferential growth of perovskite structure along a particular axis [47], which might have caused distortion of the drying particles. In other words, while the surface tension on the droplet surface tends to retain the spherical shape, the developed ionic forces in the particle during the precipitation could outweigh the surface tension force. This phenomenon is encountered in drying of other ionic solution droplets, such as NaCl, e.g., [48]. In addition, partial break down of the as-prepared perovskite particles may be responsible for the small sizes and irregular shapes of the perovskite particles.

**Fig. 4:**
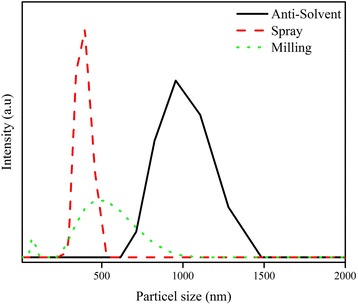
Particle size distribution of perovskite particles prepared using three different methods

**Fig. 5:**
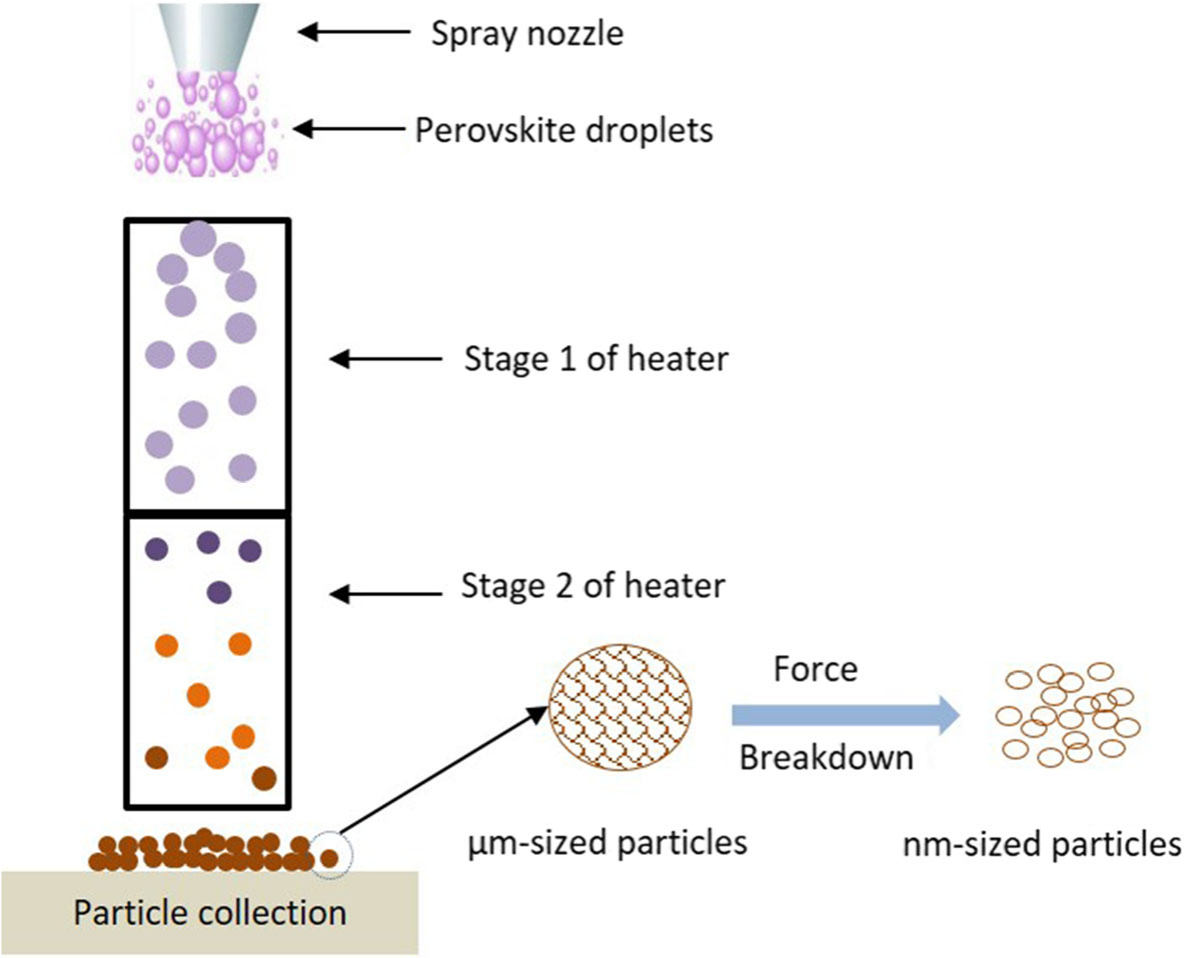
Schematic of the proposed method for the preparation of micrometer-sized and nanometer-sized perovskite particles in a suggested two-stage heater. The first stage is for rapid solvent evaporation (~ 275 °C), whereas the second stage (~ 175 °C) is for complete drying and in situ annealing

To further study the particle size, we dispersed the aforementioned powders in toluene and measured the particle size by a zeta particle sizer (Malvern, nano-zs90). Figure 4 shows the particle size distribution of the powders produced by the three aforementioned methods. Dispersion of the powders in toluene results in disintegration and breakdown of the particles, such that the individual particles have a size near or below 1 μm. This indicates that the produced particles have a weak and fragile structure and easily break down to smaller nano-sized particles. Such phenomenon has been observed by others in other particle systems, [49, 50] as well. The results also show a narrow size distribution for the particles prepared by the spray method. Based on the SEM and particle size measurements, the spray method for the preparation of the perovskite particles has been schematically shown in Fig. 5. The milling process also produces small particles, but with a wider size distribution. The powder made by the anti-solvent method has the largest particle size. Thus, the spray method produces small and mono-dispersed particles compared to the other two methods, making it a suitable method for the preparation of a perovskite paste for the deposition of thin films. The XRD patterns had already shown that the most pure and crystalline perovskite powder is obtained by the spray method as well.

## Implication of the Hypothesis

**Fig. 6:**
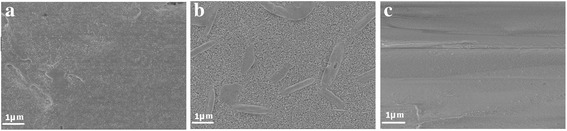
SEM images of blade-coated films from the paste of powders prepared by **a** anti-solvent, **b** milling, and **c** spray methods

**Fig. 7:**
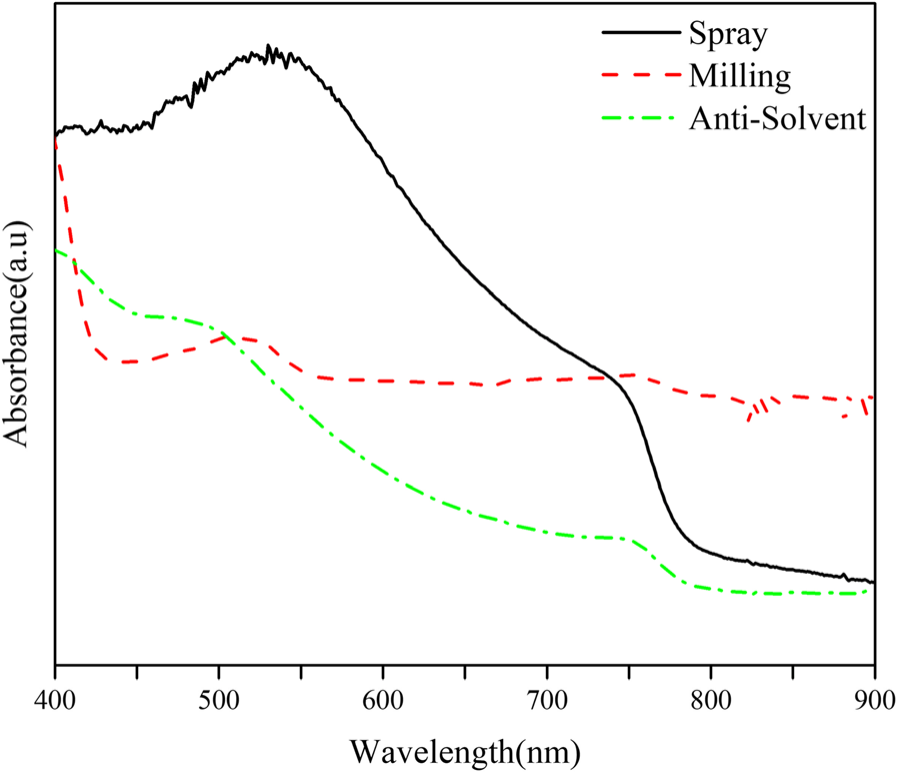
UV-Vis absorbance of the perovskite films prepared from the paste of the produced powders

We further examined the possibility of using the produced powders to prepare perovskite films. Fluorine-doped indium tin oxide (FTO)-coated glass substrates, washed with detergent, water, and isopropanol and treated with the UV radiation, was used as the starting substrate. Then, layers of compact TiO_2_ (c-TiO_2_) and m-TiO_2_ were deposited atop the FTO-coated glass, sequentially. For the c-TiO_2_ layer, 2.54 ml of titanium isopropoxide was diluted in 16.9 ml of ethanol, and 350 μl of HCl (2 M) was diluted in 16.9 ml of ethanol. The HCl solution was added to the titanium isopropoxide solution dropwise, under stirring condition, and the resulting solution was spun onto the FTO-coated glass at 2000 rpm for 60 s and annealed at 500 °C for 30 min. In order to fabricate the m-TiO_2_ layer, titanium dioxide paste diluted by ethanol (2:7 mass ratio) was spun on the c-TiO_2_ layer at 5000 rpm for 30 s and annealed at 500 °C for 30 min. Then, the perovskite paste was prepared by adding 10 μl of ethanol to 20 mg of the produced powders. The paste was deposited on the m-TiO_2_ layer at room temperature at a speed of 3 mm/s with a blade coater. The SEM images of the perovskite films are shown in Fig. 6, where it is observed that only the film deposited by the paste prepared by the spray-generated powder is uniform and fully-covered. This is partly due to the small particle size and a narrow size distribution associated with the aforementioned particles, as shown in Fig. 4. The UV-Vis absorbance (Lambda 20, Perkin Elmer Inc., USA) of the aforementioned perovskite thin films are shown in Fig. 7, where it is substantiated that the perovskite film prepared with the spray-generated perovskite powder shows a standard absorbance profile, with a sudden drop in the absorbance around the wavelength of 750 nm, which is the characteristic of the perovskites [51].

**Fig. 8:**
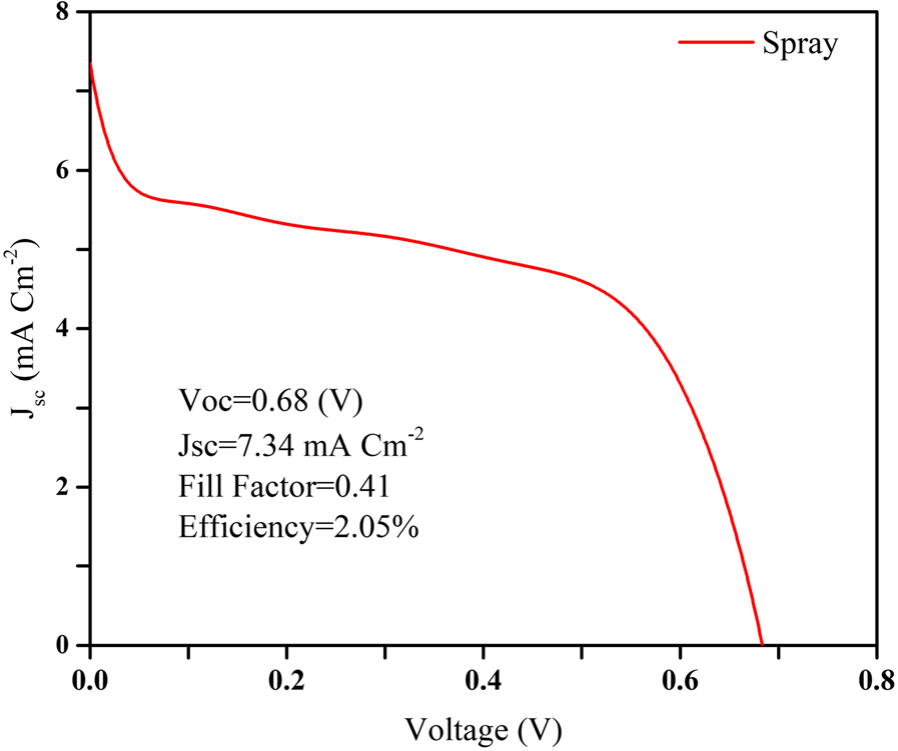
JV curve and photovoltaic parameters of a solar cell incorporating the perovskite film prepared from the spray-generated perovskite powder

In order to further test the proposed idea, a basic perovskite solar cell was fabricated, incorporating the MAPbI_3_ perovskite film prepared with the spray-made particles. To this end, spiro-OMeTAD was deposited atop the perovskite film, based on the procedure explained elsewhere [51], and then 100 nm of Au was thermally evaporated to complete the device. The JV curve and the photovoltaic parameters of the fabricated device are shown in Fig. 8. The power conversion efficiency (2.05%) is low due to low open circuit voltage (*V*_oc_), short circuit current density (*J*_sc_), and fill factor (FF). This may be mainly attributed to the insufficient binding between the perovskite particles in the film, which has presumably resulted in excessive charge recombination, due to an inadequate charge transfer from the perovskite to the adjacent layers (TiO_2_ and spiro-OMeTAD). Nevertheless, the successful fabrication of a perovskite solar cell shows the merit of the proposed method, i.e., the fabrication of the perovskite particles by spray coating. In this work, ethanol was used to prepare the paste and bind the particles. Using more suitable additives that do not dissolve the perovskites and at the same time play the role of an effective glue would improve the quality of the films and the device performance.

## Conclusions

In this work, we introduced three ideas for the preparation of perovskite particles and perovskite pastes to produce thin films. It was demonstrated that the powder prepared by spraying of the perovskite solution is crystalline and impurity-free, and has a small particle size and size distribution. Perovskite pastes and thin films were prepared using the aforementioned perovskite powders, where the perovskite film prepared using the spraying technique showed a standard morphology and light absorbance. A mesoporous perovskite solar cell was fabricated using the perovskite film prepared by the sprayed particles, where an efficiency of 2.05% was measured.
